# Analysis of factors influencing general practitioners’ decision to refer obese patients in Australia: a qualitative study

**DOI:** 10.1186/s12875-015-0262-5

**Published:** 2015-04-08

**Authors:** Kyoung Kon Kim, Lin-Lee Yeong, Ian D Caterson, Mark F Harris

**Affiliations:** Department of Family Medicine, Gachon University Gil Medical Centre, 774beon-gil 21, Namdongdae-ro, Namdong-gu, Incheon, Korea; Centre for Primary Health Care and Equity, University of New South Wales, Sydney, NSW 2052 Australia; Boden Institute, University of Sydney, Sydney, Australia

**Keywords:** GP, Obesity, Referral, Bariatric surgery

## Abstract

**Background:**

Referral for both lifestyle and surgical interventions are recommended as part of the clinical management of obesity in general practice. However, current practice falls short of this. This qualitative study aimed to describe the factors influencing general practitioners’ (GPs) referral intentions for their obese patients.

**Methods:**

Semi-structured qualitative interviews were conducted with 24 GPs from four geographically different areas in New South Wales, Australia about the management of their obese patients. A qualitative analysis was applied using inductive thematic analysis.

**Results:**

The predominant factors influencing GPs’ referral were their own attitudes and experience, and their patient’s motivation. Lifestyle intervention Referrals were usually initiated by GPs and influenced by their patients and the local health system. Referrals to conduct bariatric surgery were frequently initiated by the patient and influenced by GPs’ limited previous experience, patients’ expectations and ability to pay, as well as professional and legal issues. There was no strong link between referral and the remoteness of areas or the availability of surgical referral services.

**Conclusion:**

There were differences between GPs reported referral behaviour for lifestyle and surgical interventions. GPs’ attitudes to referral were often formed by their limited case experience rather than by a review of more systematic evidence, especially for surgical interventions. These patterns may be improved by educating and better communicating with GPs about the outcomes for their patients when they are referred.

**Electronic supplementary material:**

The online version of this article (doi:10.1186/s12875-015-0262-5) contains supplementary material, which is available to authorized users.

## Background

Obesity is an epidemic in many developed countries. Its burden on public health is substantial and increasing. In Australia, 28.3% of adults are obese (BMI ≥ 30 kg/m^2^). The prevalence of adult overweight and obesity has increased from 56.3% in 1995 to 63.4% in 2011-12 [1]. It is estimated that the total direct costs for overweight and obesity in Australia in 2005 was $21 billion and indirect costs $35.6 billion per year [2].

Obesity is common in Australian general practice patients. According to the BEACH study, the proportion of obese adults attending GPs increased from 20.9% in 2002-03 to 26.1% in 2012-13 [3]. Obesity is often difficult to manage in general practice because of a range of factors, including a high rate of relapse, lack of resources and lack of onward referral options [4]. The Australian evidence-based guidelines for the management of overweight and obesity recommend a multidisciplinary approach across the 5As (Ask, Assess, Advise/Agree, Assist and Arrange) [5-7]. These guidelines recommend behavioural interventions including referral for diet and physical activity. Where the BMI is >40 or >35 and accompanied by comorbidity, the guidelines recommend that surgical intervention be considered. Despite these recommendations, obese patients are infrequently referred by Australian GPs [8].

There is a lack of research into GPs’ decisions and intentions to refer obese patients for lifestyle programs or surgery. This paper explores the factors that influence GPs’ decision-making and intentions to refer patients by applying the theory of planned behaviour [9]. This theory has previously been applied to analyse the elements influencing the management of risk factors in general practice [8]. In this study, we hypothesized that GPs’ decisions to refer are influenced by their behavioural intentions and the primary determinants of these intentions, including attitude, subjective norms and perceived behavioural control.

## Methods

### Study design, setting and participants

A qualitative study design was chosen to explore GPs’ perspectives on obesity referral. Purposive sampling was used to recruit GPs working in primary care organisations (Medicare Locals) located in four different areas of New South Wales (NSW), Australia, with higher and lower socioeconomic patients: two in Sydney Metropolitan Area (one inner and one outer urban) and two in regional and rural NSW. GPs were invited to participate in the study via an email sent by the primary care organisations’ Local Liaison Officers. Out of 32 GPs initially identified as possibly interested in participating, 24 (75%) finally consented and were interviewed. There were 12 GPs from each of the metropolitan and regional areas. More specifically, they were located in the following areas: Illawarra-Shoalhaven, regional NSW (ISML) (n = 5); South Western Sydney, Sydney Metropolitan (SWSML) (n = 5); Western NSW, regional NSW (WNSWML) (n = 7); and South Eastern Sydney, Sydney Metropolitan (SESML) (n = 7). Although the Illawarra-Shoalhaven area is not in the Sydney Metropolitan area, GPs located there reported that the access to bariatric surgery in the area was similar to that in the Sydney metropolitan area. Two-thirds of GPs in the metropolitan area stated using both English and non-English languages during consultations. All but one of the regional area GPs reported using only English in their consultations. Table 1 provides a summary of participants’ demographics.

**Table 1:** **Demographic characteristics of participants**

**Area**		**SESML**	**SWSML**	**ISML**	**WNSWML**
**N**		**5**	**7**	**5**	**7**
**Gender**	Male	3	3	0	5
	Female	2	4	5	2
**Age**	≤39	0	0	1	1
	40-49	3	2	4	1
	50-59	1	3	0	2
	60-	1	2	0	3
**Practice size** ^*^	Single	3	1	0	0
	Small	0	2	0	1
	Medium	0	1	0	2
	Large	2	2	4	3
**Language**	English only	1	3	5	6
	English and Non-English	4	4	0	1
**Socioeconomic status of patients**	High	0	0	0	0
	Medium	1	5	3	3
	Low	4	2	2	4
**% Private health insurance** ^**†**^	0-34%	2	4	3	5
	35-69%	1	2	1	2
	70%+	0	1	0	0
**Distance from bariatric surgery**	0-14 km	4	7	3	1
	15-49 km	1	0	2	0
	50-99 km	0	0	0	1
	100-199 km	0	0	0	2
	200 km-	0	0	0	3

*Abbreviation:* SESML, South East Sydney Medicare Local; SWSML, South Western Sydney Medicare Local; ISML, Illawarra-Shoalhaven Medicare Local; WNSWML, Western New South Wales Medicare Local.

SESML and SWSML are in Sydney metropolitan area.

ISML and WNSWML are classified as regional areas.

^*^Data of three participants are missing.

^†^Data of three participants are missing.

### Data collection

Data was collected between November 2013 and July 2014 using semi-structured interviews. Six main topic areas were covered in the interviews: experience, initiation, opinion, prioritising, cost, and waiting time for referral to lifestyle and surgical weight-loss options and scenarios (Additional file 1). Each topic area included between 2 to 6 mostly open-ended questions to facilitate a guided exploration of GPs’ perspectives. Interviews were conducted in English by four interviewers trained in qualitative techniques and guided by the research team beforehand. Interviews lasted 20 to 60 minutes. They were recorded and then transcribed verbatim.

### Data analysis

Data was analysed using a mixed-method approach. Transcripts were thematically coded using TAMS Analyzer (version 4.47b4ahMav, Boston, USA) and manually. Data was mapped to emergent themes and subthemes. Data was declared to have reached saturation once no new themes were emerging. Data was analysed by researchers Kim, Yeong and Harris and their interpretation verified during multiple research meetings where emerging crosscutting themes were formulated and discussed.

### Ethics

Approval to conduct this study was granted by the University of New South Wales’ Human Research Ethics Committee. All participants gave their informed consent. In reporting the results we have assigned a number for each participants and a geographic identifier to ensure anonymity.

## Results

There was variability in the referral rates of obese patients to bariatric surgery reported by the GPs’ interviewed – between 0 and 10 patients over the previous 12 months. Referrals to weight-loss lifestyle programs or allied health providers, such as dieticians and exercise physiologists, were more frequent than for bariatric surgery.

### Factors influencing GPs’ decision to refer to lifestyle interventions

Referral was frequently made to individual providers (such as private dieticians, exercise physiologists or endocrinologists). A few GPs reported referring to specialist obesity clinics in hospitals. Many GPs reported that they, rather than patients, initiated referrals to lifestyle intervention. Also, GPs reported little influence of patients’ professional or medico-legal expectations.

**Figure 1:**
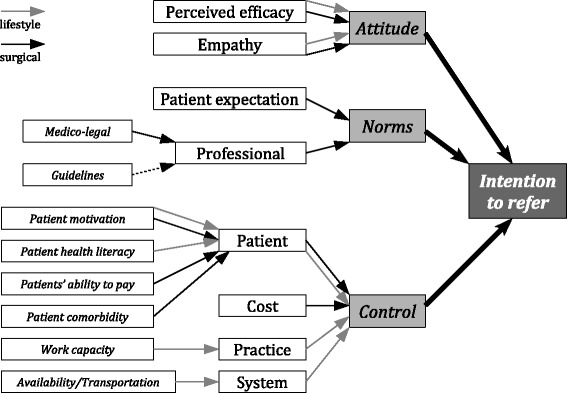
**Analysis of intentions to refer using theory of planned behaviour.** The grey arrow is for influence on referrals of non-surgical intervention, and the black arrow is for surgical intervention. The dotted line is for weak influence.

GPs intentions to refer patients for lifestyle interventions were mostly influenced by their own attitudes and external factors, such as patients’ motivation, health literacy, ability to pay, comorbidity, cost, work capacity and availability of resources (Figure 1). Their belief, or lack of it, in the effectiveness of the referred intervention in helping patient change their behaviour in order to lose and maintain weight also influenced their referral intentions.

*So I think the main problem with referral is that it just doesn’t actually change behaviour, which is the main issue, I think, with dealing with overweight people. [Rural GP #24]*

*On the whole I’d say the success rate is quite low, in terms of major changes. [Urban GP #2]*

The GPs beliefs about effectiveness were largely based on anecdotal feedback from patients previously referred.

*Most of them go and say, “I didn’t really learn anything I didn’t already know.”[Rural GP #24]*

There appeared to have been little direct communication between the GPs and the referral services or providers.

*We’ve got a good dietician in town and he sits down and talks to them with a combination of regular exercise program and diet and only a tiny percentage of people lose weight. [Rural GP #12]*

*If people go to the public system, it’s a black hole. … They just disappear and we don’t even know if they get there or what the outcomes are. [Rural GP #11]*

Patient motivation and health literacy exerted a strong influence on the GPs’ decision to refer:

*I want lots of people with a BMI over 30 to go somewhere, but most are not really interested or motivated to change. [Rural GP #1]*

*…they may or may not put changes in place. But again, motivation is probably the biggest issue there.” [Urban GP #7]*

A few GPs reported that their experience with managing their own weight influenced their approach to referral.

Practice nurse capacity and the distance and availability of transport to reach referral services also influenced GPs’ decision to refer. Where services were available within the practices or at least in nearby in rural towns, GPs reported being more ready to refer.

*We've got the facility of the dietitians who have been coming over here once a fortnight from the division and they have been really excellent. [Urban GP #9]*

*So dietitians are in town and easy to get into, well fairly easy, certainly easy to refer to. [Rural GP #11]*

### Factors influencing GPs’ decision to refer for bariatric surgery

#### Attitudes and options

GPs had a range of attitudes towards bariatric surgery. At one end, a few GPs reported that they seldom referred patients, as they believed it was rarely of value and were very pessimistic about how successful this method was. Some felt it was a last resort, because of the cost and risks associated with it.

*I wouldn’t refer someone for bariatric surgery if I didn’t think that they’d adequately explore [other] options. [Rural GP #7]*

*It is a last resort. [Urban GP #3]*

At the other end, other GPs felt it was often successful and was really the only option where major weight-loss was required.

*If they are only 30 to 32 they might improve. But if BMI is 40 plus, [lifestyle] interventions aren’t strong enough. [Urban GP #18]*

In contrast with referrals to lifestyle programs, GPs’ decision to refer patients for a bariatric surgery was strongly influenced by their patients’ expectation or request. A number of GPs reported that patients were actively seeking surgery when they were motivated and financially ready. Consequently, most referrals were based on patients’ request or decision.

*They want it [referral for bariatric surgery] more than we want to do it. [Urban GP #23]*

*So it’s [the subject of bariatric surgery] often generated quite early on from the patient. [Rural GP #16]*

Some GPs reported that their patients’ attitudes and beliefs about surgery were a barrier to referral.

*There is a lot of stigma; a lot of patients are embarrassed to be referred for surgery. [Urban GP #19]*

Further, some GPs felt that their patients’ requests for surgery were based on assumptions about surgery being an easier option.

*A lot of people view it as an easy option. [Rural GP #4]*

*I think often that sort of feeling that, this will be a quick fix, and that it will be easy and it’s not easy and it still takes quite a lot of discipline. [Urban GP #2]*

GPs often expressed negative attitudes towards these patients. These attitudes were mostly informed by past experiences with a relatively small number of patients – who had regained weight or experienced serious side effects following the surgery.

*One of them just didn’t lose any weight, and she’s actually moved out of the area, so I lost the follow-up. Another one had a lot of serious life threatening complications which are still going on. [Rural GP #4]*

*Nowadays he does gastric banding left right and centre and the follow-up is not, unfortunately, as good as before and the outcome is not that good. The patient gets around the gastric banding by eating small amounts of a high calorie food … [A]s I said before, one out of 10 they maintain their weight, the rest, nine, they go back to what they were. And the discomfort in the stomach is so bad and ultimately we have to remove the gastric banding. After spending a lot of money it is a real sheer waste of time. [Rural GP #12]*

GPs that were positive about bariatric surgery, and continued to actively refer patients, were often influenced by positive feedback from patients. GPs considered the value of surgery to be in its contribution to long-term maintenance of major weight-loss even though this might or might not have been achieved. Some were influenced by the side effects reported by previous patients:

*… it got pretty spectacular results but it was a cow of a process to go through. Like really, really uncomfortable …[Urban GP #9]*

*One had to have it [band] removed, ‘cause it moved and caused infections and problems. [Rural GP #15]*

They were more positive about bariatric surgery if they believed the surgeons were trustworthy and had a competent multidisciplinary team.

*We also recognise that it needs to be a multidisciplinary-type team that actually both assesses people to be done and supports them through the procedure, that it isn’t just, do the surgery, fix and forget. [Rural GP #2]*

GPs who felt they had a variety of options were more likely to refer patients to surgical or lifestyle services regardless of their negative attitude towards bariatric surgery.

#### External factors

Although many referrals were patient-driven, a number of GPs recognised the value of surgery, especially for their patients with comorbidities. This was based on the GPs’ clinical assessment of individual patients and, while cost, access and feedback from previous patient experiences were accounted for, these GPs reported referring patients they believed would benefit from surgery. This was based on experience and guidance and guidelines from professional bodies. A few GPs reported being influenced by medico-legal considerations.

Across the four Medicare Locals, GPs considered cost to be major factor influencing their decision to refer for bariatric surgery or not.

*Usually I wait for them to ask, mainly because it's a personal thing because of the course, because I know it's not really accessible in the public system. So I'm very sensitive. I don't want to offer something that is not accessible to them if they don't have a private health fund or if there is no superannuation that they can tap into. [Urban GP #10]*

*Cost is another barrier… it’s not done under the public system, but people have to find somewhere up to $10,000 to have this surgery done. And so that basically excludes the lowest classes of people who might need it most because they have high risk and comorbidity. [Rural GP #3]*

Surprisingly, there was no strong relationship between this variability in reported referral and availability of services, with GPs in the more remote areas (WNSWML and ISML) reporting that they were no less likely to refer patients to surgery or to lifestyle programs or providers. In fact, GPs from ISML felt they had a variety of options and were most likely to report referring patients to bariatric surgery and to allied health and weight-loss programs. GPs from SWSML were more likely to report limited access and were less likely to refer patients to surgical and lifestyle services.

All GPs reported that surgery was virtually unavailable in public hospitals.

*I actually haven’t had any patients that have had this surgery done under the public system. [Rural GP #13]*

*I’m unaware of public bariatric surgery. [Urban GP #19]*

When looking at languages used during consultation, there was no marked difference between GPs who consulted in English and GPs who consulted in another language. Patients’ main language was not reported by the GPs to be a strong factor influencing referral. However, when looking at low-income populations, mostly residents of South Western Sydney area from migrant backgrounds, GPs were more likely to report difficulties making referral.

*There's nowhere to send. Where do you send them? Dietitian, you have to have other private insurance. If you have no private insurance it become public hospital, and already public hospital already put a lot of restrictions really and long waiting period for these people. [Urban GP #8]*

### GPs’ involvement in the lead and follow-up of bariatric surgery

GPs either prepared patients before surgery or made the referral and expected the surgical team to provide multidisciplinary support for patients, from pre-operative assessment to long-term follow-up.

GPs who perceived their patient’s psychological state to be a major factor influencing the effectiveness of surgery were more likely to take time – often months – to assess their patients’ eligibility for surgery and ability to handle it psychologically and physically.

*I felt personally that I needed to take a very sort of active role in trying to make sure that if I was referring someone that I felt that they had had other support mechanisms in place… it’s very much the psychosocial side of how that affects your life and I don’t think people always appreciate that. [Urban GP #2]*

Other GPs who were more likely to rely on surgical multidisciplinary teams reported access to effective surgical teams with reliable pre-operative assessment and post-surgical psychological and physical follow-up.

*[Hospital for bariatric surgery] got a whole system, there’s the dietitians and I think they see a psychologist and there’s a whole team approach. [Urban GP #19]*

## Discussion

Most GPs were ready to refer obese patients for lifestyle interventions with most reporting that they usually initiated the discussion of referral options. This is despite the evidence that referral rates from general practice for educational or behavioural interventions are low in Australian general practice overall [10,11]. The predominant factors influencing GPs’ referral were their attitudes and perception of how motivated and literate patients were in relation to their health. Many GPs were concerned about the effectiveness of lifestyle interventions in achieving and sustaining weight-loss. Logistical factors, such as the availability of and distance to referral services, also had some influence, but were not major barriers. GPs reported being less influenced by professional standards such as the National Health and Medical Research Council guidelines [5] or patient expectations. These findings are broadly consistent with previous research [12-14].

By contrast with lifestyle referrals, GPs in this study expressed ambivalence towards surgery. They only very infrequently reported referring obese patients for surgical interventions. When they did, it was often because previous interventions had failed and consistent with the guidelines pertaining to the threshold for referral (BMI > 40 or BMI > 35 with comorbidities). However, this type of referral was most commonly initiated by the patient rather than by the GP, which is not consistent with the approach outlined in the guidelines or the evidence. Reasons for this reluctance to initiate this type of referral included concerns about the ability of the patient to pay, patients reacting negatively to the suggestion, and previous negative experience with complications or failures. GPs’ attitudes were strongly influenced by their often very limited experience and feedback from patients. It is possible that this might have been compounded by GPs’ lack of knowledge about the positive outcomes discussed in long-term follow-up studies [15]. GPs considered professional norms and legal responsibilities, costs of surgery and the financial situation of patients when deciding whether to refer their patients to surgery or not.

The GPs’ intentions can be understood within the framework of the theory of planned behaviour with factors having more or less weight in referral intentions for surgical and lifestyle interventions (see Figure 1) [9,16]. The pattern of influences was different for each type of referral. Attitudes and external factors, such as availability of local services and transport, and patient motivation, significantly influenced GPs’ intentions to refer patients for lifestyle interventions. GPs’ intentions to refer for surgery were influenced by their attitudes about efficacy, normative influences – including patient expectations and legal requirements – and control factors –especially cost and patient comorbidities.

We have been able to find relatively few other studies that explored reasons for surgical and lifestyle referrals from general practice. A recent study in New Zealand also identified stigma, concern about effectiveness of interventions and availability of local services as barriers to GP referral [12,17].

These findings have implications for health policy and practice in Australia. GPs’ attitudes were a barrier to both surgical and lifestyle referral and were often based on limited case experience (especially for surgical interventions) rather than a review of the evidence. These attitudes may be improved not only by educating, but also by providing better feedback to GPs about the outcomes of interventions to which their patients are referred. Surprisingly few GPs reported practice or local systems as being major barriers to deciding on either type of referral. However, co-located providers or local availability facilitated lifestyle referral. This suggests that GPs may be amenable to information education and improved communication along the referral pathways by trusted primary care organisations. Cost was of course a major barrier with most GPs stating that there was very little or no access to publicly funded surgery.

### Study limitations

Though the distribution of the participating GPs across the four Medicare Locals and the saturation of themes are strengths of the study, the fact that this research was carried out in only one state of Australia means that these findings cannot necessarily be generalised to all Australian GPs. The potential limitation relating to interview and analysis bias was reduced by the use of well trained and experienced interviewers and having the coding and analysis conducted by three investigators independently with differences resolved in multiple meetings and discussions.

## Conclusions

Although many GPs were concerned about their effectiveness in achieving sustained weight-loss, most were ready to refer obese patients for lifestyle interventions and only very infrequently referred patients for bariatric surgery. GPs’ attitudes about these types of referrals were often formed on limited case experience, especially for surgical interventions. Further, cost and ability were major barriers, with most GPs perceiving that there was very little or no access to publicly funded surgery.

These referral practices could be improved by local health services educating GPs about the outcomes of these respective referrals or providing information encouraging them to follow guidelines about whether to refer to lifestyle programs or surgery [5]. By providing training opportunities for GPs, local health services may be able to help improve their relationship with GPs as well as give better feedback on the outcomes of lifestyle and surgical interventions. In turn, this will help shape future practice where patients can receive better care for obesity and comorbidities.

## Additional file

Additional file 1:
**Interview questions.**
