# Supplementary material for: Analysis of factors influencing general practitioners’ decision to refer obese patients in Australia: a qualitative study
Source: BMC Fam Pract. 2015 Apr 8;16:45. doi: 10.1186/s12875-015-0262-5 (PMC4396120; doi:10.1186/s12875-015-0262-5)
Supplement: Additional file 1: — Interview questions. [file 12875_2015_262_MOESM1_ESM.pdf]

Appendix 1. Interview questions

<u>Introduction:</u> This interview is to help us to understand the referral pathway for patients with a BMI of over 30 and the factors that may influence you in making decisions about where or when to refer. The interview will be recorded for the purposes of transcribing. Once we have completed the transcription and analysis the recording will be deleted. Are you happy to proceed?

1. Can you tell us about your experiences of referring patients with a BMI of over 30 to dieticians, psychologist, commercial or other medical obesity treatment programs (not involving surgery)? What is your opinion about these programs (cost, accessibility, effectiveness)?

2. Can you tell us a bit about your experience of referring obese patients for bariatric surgery? Who usually initiates the discussion (patient or yourself)? In the case where you raise it how often does the patient agree? About how many patients have you referred for obesity surgery over the last year? What proportion would this be of those who you would be suitable for referral? How do you prioritise which patients to refer? Is it different for private and public patients?

3. Where the services are available, what are the main factors that influence your decision to refer? How often do these factors influence your decision?

4. What are the costs to patients associated with private referrals for obesity management? For those with and without private health insurance? For patients with private health insurance, do costs vary across policies and insurance providers?

5. How long do patients who have been referred to above have to wait to be seen by a specialist in the public sector, what is their likelihood of being offered surgery, and if offered surgery, how long do they have to wait?

6. Consider the following scenarios:

    a. Would you consider referring a 45 year old female patient with a BMI of 41 and who has hypertension but no other health problems but who has been unable to lose weight? Would you consider very low calorie diets for weight loss? What referral options would you consider? If no, how would you manage her?

    b. Would you consider referring a 30 year old male patient with a BMI of 50 who has not been able to lose weight? Would you consider medications for weight loss? What referral options would you consider? What if he did not have private health insurance?

    c. Would you consider referring a 50 year old woman with a BMI of 33 who has osteoarthritis of the knees and type 2 diabetes and has been unable to lose

weight? What referral options would you consider? What are the likely barriers for you and for the patient? What if she did not have health insurance?
